# The use of a direct bronchial challenge test in primary care to diagnose asthma

**DOI:** 10.1038/s41533-020-00202-y

**Published:** 2020-10-16

**Authors:** J. E. Bins, E. I. Metting, J. B. Muilwijk-Kroes, J. W. H. Kocks, J. C. C. M. in ’t Veen

**Affiliations:** 1 Department of Respiratory Medicine, STZ Center of Excellence for Asthma, COPD and Respiratory Allergy, Franciscus Gasthuis & Vlietland, Rotterdam, The Netherlands; 2 Department of General Practice, University of Groningen, University Medical Center Groningen, Groningen, The Netherlands; 3 GRIAC Research Institute, University Medical Center Groningen, University of Groningen, Groningen, The Netherlands; 4 Faculty of Economics and Business, University of Groningen, Groningen, The Netherlands; 5 Star-shl, Medical Diagnostic Center, Rotterdam, The Netherlands; 6 General Practitioners Research Institute, Groningen, The Netherlands; 7 Observational and Pragmatic Research Institute, Singapore, Singapore; 8 Department of Respiratory Medicine, Erasmus MC, Rotterdam, The Netherlands

**Keywords:** Diagnosis, Asthma

## Abstract

Many asthmatics in primary care have mild symptoms and lack airflow obstruction. If variable expiratory airflow limitation cannot be determined by spirometry or peak expiratory flow, despite a history of respiratory symptoms, a positive bronchial challenge test (BCT) can confirm the diagnosis of asthma. However, BCT is traditionally performed in secondary care. In this observational real-life study, we retrospectively analyze 5-year data of a primary care diagnostic center carrying out BCT by histamine provocation. In total, 998 primary care patients aged ≥16 years underwent BCT, without any adverse events reported. To explore diagnostic accuracy, we examine 584 patients with a high pretest probability of asthma. Fifty-seven percent of these patients have a positive BCT result and can be accurately diagnosed with asthma. Our real-life data show BCT is safe and feasible in a suitably equipped primary care diagnostic center. Furthermore, it could potentially reduce diagnostic referrals to secondary care.

## Introduction

Asthma is a one of the most common chronic conditions to be dealt with in primary care. Worldwide it is estimated to affect 235 million people, with a prevalence ranging from 1 to 18% in different countries^1,2^. According to recent data, 5.7% of the Dutch population suffer from asthma^3^. Despite its high prevalence, asthma can be challenging to diagnose, especially given that most cases are diagnosed in primary care. This difficulty lies in the variable nature of key features over time, such as airflow obstruction and airway inflammation.

A diagnosis of asthma is made based on characteristic respiratory symptoms—wheeze, shortness of breath, chest tightness—and confirmed variable expiratory airflow limitation^2^. However, many people with asthma, especially in primary care, have mild symptoms and lack airflow obstruction^4^. Misdiagnosis (both underdiagnosis and overdiagnosis) is therefore a problem for both adults and children^5,6^. A factor that contributes to the misdiagnosis of asthma is the poor diagnostic sensitivity of spirometry, which cannot exclude asthma without further investigation when results are inconclusive^7^. If variability in expiratory airflow limitation cannot be determined by spirometry or peak expiratory flow, but there is a history of variable chronic respiratory symptoms, a positive bronchial challenge test (BCT) can be used to confirm the diagnosis of asthma^2,8^. However, currently in primary care settings, patients with normal spirometry results but suspected asthma must be referred to secondary care for BCT when the diagnosis remains unclear^2^. In the Netherlands, the Star-shl Medical Diagnostic Centre in Rotterdam is the only primary care diagnostic center to carry out direct BCTs by histamine provocation.

The Global Initiative for Asthma provides the most widely used strategy for the management of asthma. This suggests referral for BCT to assess airway hyperresponsiveness when variable airflow limitation—the key part of diagnosing asthma—cannot be determined^2^. In the United Kingdom, there has been debate about conflicting guidelines on the diagnosis and treatment of asthma^9^. The National Institute for Health and Care Excellence guideline advises additional testing with fractional exhaled nitric oxide (FeNO) or BCT in cases of suspected asthma with normal spirometry results^10^. However, the British Thoracic Society (BTS)/Scottish Intercollegiate Guideline Network (SIGN) guideline states that asthma is a clinical diagnosis that can be made based on a typical history, without requiring a definitive diagnostic test^11^. In contrast to these, the Dutch General Practitioner Society (Nederlands Huisartsen Genootschap [NHG]) advocates referral for a direct provocation test or for consultation with a pulmonologist when diagnostic uncertainty remains and, conflicting with the BTS/SIGN guideline, clearly stating that inhaled corticosteroid (ICS) therapy is only indicated when the diagnosis is certain^12^. None of these guidelines gives recommendations for performing BCT outside of a specialty care facility^13^.

Histamine and methacholine are the most used agents for “direct” pharmacological BCT. Most published studies are based on provocation with methacholine. The literature on histamine use is scarce. The understandable fear of possible severe bronchoconstriction has led to most authorities recommending that BCTs should only be performed in specialist care facilities. However, to date, thousands of methacholine challenge tests have been performed without serious side effects^14^. Transient and mild symptoms are common in patients with bronchial hyperresponsiveness (BHR), including symptoms of wheeze, cough, dyspnea, and chest tightness, though many experience no symptoms. Moreover, delayed or prolonged responses to methacholine are rare^8^.

Direct BCT is used to increase or decrease the diagnostic probability of asthma by determining if BHR is present at that time. BHR is not an exclusive feature of asthma and can also present in chronic obstructive pulmonary disease (COPD), respiratory infections, and allergic rhinitis^15^. In general, testing for BHR has high sensitivity and low specificity meaning that every BCT needs to be appropriately interpreted, taking into account not only the test result but also the presenting symptoms and history^16^. However, in patients with a high pretest probability of asthma, a negative test result rules out asthma and a positive test result can confirm the diagnosis of asthma^8^. In addition, the probability that a positive BCT reflects asthma will increase the lower the PD_20_ (provocative dose causing a 20% decline in forced expiratory volume in 1 s (FEV_1_) from baseline)^17^.

In this observational real-life study, we primarily aimed to evaluate the feasibility and safety of direct BCT in a Dutch primary care diagnostic center. We had two secondary aims: (1) to analyze the characteristics of patients with a high probability of asthma and to identify any predictors of a positive BCT in this group and (2) to explore whether using BCT in primary care has the potential to reduce diagnostic referrals to secondary care and the number of asthma misdiagnoses.

## Results

### Feasibility and safety

In total, 998 patients (*n* = 640 female, 64.1%) were identified who had undergone a histamine provocation test at the diagnostic center, equating to an average of 200 patients a year; among these, BCT was deemed “not assessable” in 8 patients (<1%).

Throughout the retrospective study period, there were no adverse events during BCT, and there was never a reason to consult a doctor during or directly after BCT for such concerns. Although the post-BCT FEV_1_ was required to return to ≥90% of baseline, necessitating that bronchodilators were administered when needed, only 4 patients (<1%) needed a bronchodilating agent in addition to the salbutamol to recover to their baseline FEV_1_. In each of these cases, the addition of ipratropium bromide was enough to bring about recovery and allow them to go home safely. Patients were advised to contact their general practitioner (GP) whenever they would experience symptoms after leaving the diagnostic center after the BCT. There were no GP consultations or emergency department visits reported back to us. We did not have direct access to data from these services, but as there is a close connection between the diagnostic center and the referring GPs, we find it very unlikely that in this 5-year time span any serious side effects have taken place without us knowing.

Almost two-thirds of the total cohort (*n* = 645) had undergone spirometry at the diagnostic center, including bronchodilator testing, prior to BCT. The baseline characteristics of this final cohort (*n* = 645) who underwent both spirometry and BCT at the diagnostic center are shown in Table 1.

**Table 1:** Baseline characteristics of patients undergoing spirometry with bronchodilator response and BCT at the diagnostic center (*N* = 645).

Variable	Results	Missing data
Age, mean ± SD (range)	43.24 ± 14.87 (16–76) years	*n* = 13
Age of onset, mean ± SD (range)	33.3 ± 18.38 (0–71) years	
Sex, *n* (%)		
Male	231 (36%)	
Female	414 (64%)	
BMI, mean ± SD (range)	27.8 ± 5.52 (16–53) kg/m^2^	*n* = 1
Smoking		
Never	359 (56%)	
Previous	195 (30%)	
Current	91 (14%)	
Family history (asthma), *n* (%)		
Positive	278 (44%)	
Negative	291 (46%)	
Unknown	76 (10%)	
ACQ total, mean ± SD (range)	1.37 ± 0.90 (0–4.83)	
FEV_1_ in L, mean ± SD (range)		
Pretest, L	3.08 ± 0.807 L (1.49–5.96 L)	*n* = 12
% predicted	95.85% ± 13.04% (67.5%–141.6%)	*n* = 13
Post-test, L	3.18 ± 0.84 L (1.62–6.17 L)	*n* = 1
% predicted	99.02% ± 13.14% (66–139%)	*n* = 1
FEV_1_/FVC %, mean ± SD (range)		
Pre-BD	78.49 ± 7.80 (54.70–100)	*n* = 12
Post-BD	81.61 ± 7.49 (54.45–100)	
FEV_1_/FVC ≤ 70%, *n* (%)		
Pre-BD	85 (13%)	
Post-BD	43 (7%)	
Reversibility %, mean ± SD (range)	3.55% ± 4.0%	*n* = 12
10–12%	16	
≥12%	4	
Reversibility in mL, in case of ≥12%	372 ± 49.92 (320–440)	
ICS use, *n* (%)		
ICS mono	84 (13.0%)	
ICS/LABA	133 (20.6%)	
ICS total	217 (33.6%)	
Antihistamine use, *n* (%)		
Oral	81 (12.5%)	
Nasal	55 (8.5%)	
Ocular	3 (0.5%)	
Self-reported allergy, *n* (%)		
Pets	76 (12%)	
Food	26 (4%)	
Hay fever/seasonal	141 (22%)	
Wheezing (ACQ5), *n* (%)		
Yes	392 (61%)	
No	253 (39%)	
AB or prednisone ≥1 course last year, *n* (%)		
No	449 (70%)	
Yes	191 (30%)	
Working diagnosis from spirometry, *n* (%)		
Probable asthma	363 (56%)	
Possible asthma (unclear)	221 (34%)	
Asthma	27 (4%)	
COPD	14 (2%)	
Asthma/COPD	8 (1%)	
No airflow obstruction	6 (1%)	
Possible restriction	3 (0.5%)	
Poor curve	3 (0.5%)	

*AB* antibiotics, *ACQ* Asthma Control Questionnaire, *BD* bronchodilator, *BMI* body mass index, *COPD* chronic obstructive pulmonary disease, *FEV*_*1*_ forced expiratory volume in 1 s, *FVC* forced vital capacity, *ICS* inhalation corticosteroid, *LABA* long-acting beta agonist, *SD* standard deviation.

The indication for BCT was accurate in most patients, yet in 41 patients (6%) the indication for BCT was debatable. Based on the results of spirometry and history, 27 patients already diagnosed with asthma were referred for BCT. Most of these patients showed borderline reversibility, but 4 showed reversibility of ≥12% and ≥200 mL and it is unclear why these were referred for BCT. Another 14 patients were diagnosed with COPD before referral for BCT. The indication for a BCT was not reported in these patients; we assume asthma remained in the differential diagnosis due to a fixed obstruction at spirometry.

### Analysis of patients with high probability of asthma

**Fig. 1:**
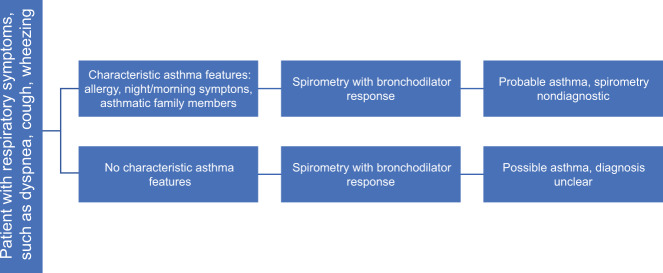
Probable asthma and possible asthma groups. Flowchart showing how the probable asthma and possible asthma groups are organized.

Histamine provocation testing was positive in 376 (58%) of the included participants in our final cohort of 645 patients (Table 1), which was similar to the percentage in the overall study population (574/998, 58%). All patients were given a working diagnosis after spirometry, with 363 (56%) labeled as probable asthma and 221 (32%) labeled as possible asthma (Fig. 1). These 584 patients comprised the cohort with a high pretest probability of asthma. The details of the probable and possible asthma groups are compared in Table 2.

**Table 2:** Possible asthma and probable asthma patient characteristics.

Variable	Possible asthma, *N* = 221	Probable asthma, *N* = 363	*P* value^a^
Age, mean ± SD (range)	46.01 ± 14.35 (16–76)	41.04 ± 14.85 (16–71)	0
Age of onset, mean ± SD (range)	37.81 ± 18.64 (0–70)	30.98 ± 17.46 (0–71)	0
Sex, *n* (%)			
Male	87 (39%)	120 (33%)	0.122
Female	134 (61%)	243 (67%)	
BMI, mean ± SD	27.798 ± 4.99 kg/m^2^	28.03 ± 5.95 kg/m^2^	0.626
Smoking, *n* (%)			0.662
Never	131 (59%)	206 (57%)	
Previous	64 (29%)	105 (29%)	
Current	26 (12%)	52 (14%)	
Medication use, *n* (%)			0.908
ICS mono	21 (10%)	52 (14%)	
ICS/LABA	35 (16%)	75 (21%)	
ICS total	56 (25%)	127 (35%)	
Reversibility, mean ± SD	2.84% ± 3.39%	3.66% ± 4.03%	0.013
FEV_1_/FVC %, mean ± SD (range)			
Pre-BD	78.46 ± 7.82 (54.70–99.47)	79.35 ± 7.12 (61.42–100)	0.179
Post-BD	81.11 ± 7.50 (55.83–99.41)	82.66 ± 6.58 (60.13–100)	0.016
FEV_1_/FVC ≤ 70%, *n* (%)			
Pre-BD	27 (12%)	32 (9%)	0.286
Post-BD	13 (6%)	9 (2%)	
Antihistamine use, *n* (%)			0.219
Oral	17 (8%)	57 (16%)	
Nasal	14 (6%)	36 (10%)	
Ocular	2 (1%)	1 (0.3%)	
Self-reported allergy, *n* (%)			
Pets	16 (7%)	52 (14%)	0.01
Food	7 (3%)	19 (5%)	0.24
Hay fever/seasonal	34 (15%)	90 (25%)	0.007
Wheezing (ACQ5)			0.000
Yes	110 (50%)	244(67%)	
No	111 (50%)	119 (33%)	
ACQ total, mean ± SD	1.1531 ± 0.87	1.52 ± 0.90	0.000
ACQ 1_night_	0.95 ± 1.21	1.40 ± 1.45	0.000
ACQ 2_morning_	1.30 ± 1.42	1.62 ± 1.38	0.000
ACQ 3_activities_	1.55 ± 1.18	1.93 ± 1.29	0.003
ACQ 4_dyspnea_	1.73 ± 1.31	2.25 ± 1.28	0.001
ACQ 5_wheeze_	1.08 ± 1.34	1.49 ± 1.42	0.000
ACQ 6_med_	0.30 ± 0.71	0.45 ± 0.84	0.016
AB/prednisone ≥1 course last year, *n* (%)			0.215
Yes	74 (34%)	104 (29%)	
No	145 (66%)	256 (71%)	
Exacerbation/last year, *n* (%)			0.3
0	160 (72%)	275 (76%)	
1	40 (18%)	48 (13%)	
≥2	21 (10%)	40 (11%)	

*AB* antibiotics, *ACQ* Asthma Control Questionnaire, *BD* bronchodilator, *BMI* body mass index, *COPD* chronic obstructive pulmonary disease, *FEV*_*1*_ forced expiratory volume in 1 s, *FVC* forced vital capacity, *ICS* inhalation corticosteroid, *LABA* long-acting beta agonist, *SD* standard deviation.

^a^For comparing the two groups, we used *t* test for normally distributed continuous variables or Mann–Whitney *U* test for continuous variables not normally distributed. Chi-square test was used for nominal variables.

Compared to the possible asthma group, those with probable asthma were significantly younger (mean 41.04 ± 14.5 vs 46.01 ± 14.35 years, *P* < 0.001, Mann–Whitney *U*) and had an earlier symptom onset (mean 30.98 ± 17.46 vs 37.81 ± 18.64, *P* < 0.001, Mann–Whitney *U*). The probable asthma group also had significantly higher mean reversibility after bronchodilation (BD; FEV_1_ 3.66% ± 4.03% vs 2.84% ± 3.39%, *P* = 0.013, *t* test) and higher mean scores on the Asthma Control Questionnaire (ACQ; 1.52 ± 0.90 vs 1.15 ± 0.87, *P* < 0.001, Mann–Whitney *U*). Self-reported allergies for pets and pollen were higher in the probable asthma group (*P* = 0.010 and *P* = 0.007 respectively, and *χ*^2^). These significant differences underpin why the probable asthma group is more likely to have asthma than the possible asthma group.

In the probable asthma group, 223 patients (61.4%) experienced bronchial hyperreactivity during BCT and were consequently accurately diagnosed with asthma, while the absence of hyperreactivity excluded asthma in the remaining 140 patients. In the possible asthma group, another 110 patients (49.8%) had a positive BCT result and were diagnosed with asthma, while the diagnosis was excluded in the other half of this population.

Airflow obstruction post BD (FEV_1_/forced vital capacity (FVC) post BD ≤70%) was detected in 43 patients (7%) of the final cohort, suggesting either COPD or a fixed obstruction in asthma. Of these patients, 72.1% (*n* = 31) had a positive BCT result. In the group with a high pretest probability of asthma, 14 of the 22 patients (possible asthma = 13; probable asthma = 9) with airflow obstruction post BD had a positive BCT result (64%).

A total of 217 patients were already using ICS when performing BCT. Despite treatment with ICS, 77% (*n* = 133) of these patients still had a positive BCT result. In the high probability group, this percentage was lower; of the 183 patients on ICS, 103 patients (56%) had a positive test result.

When comparing all patients by BCT result, there were few significant differences between the 333 patients with positive results (probable asthma = 223; possible asthma = 110) and the 251 patients with negative results (probable asthma = 140; possible asthma = 111). Reversibility after BD was significantly higher in the group with positive BCT results (mean 3.73% ± 3.94%) than in the group with negative BCT results (2.85% ± 3.60%, *P* = 0.006, *t* test). Although patients were slightly younger in the positive BCT group (41.92 years) than in the negative BCT group (44.24 years), the difference was not significant (*P* = 0.069, Mann–Whitney *U*). Similarly, there was a non-significant difference in question 6 on the ACQ (extra bronchodilator use), with the positive BCT group scoring higher (mean 0.46 vs 0.31, *P* = 0.079, Mann–Whitney *U*).

### Diagnostic accuracy and diagnostic referrals

All 363 patients with probable asthma had typical histories of asthma, but only 223 (61.4%) had bronchial hyperreactivity proven by BCT. Half of the 221 patients with possible asthma had a positive BCT, confirming asthma. In 251 patients with probable and possible asthma, a negative BCT result meant that asthma could be excluded (251/584, 43%).

Diagnostic referral to secondary care in this cohort could have potentially been reduced by at least 333 patients (57%, 333/584) who had a positive test confirming the diagnosis of asthma. Patients with a negative test may still need referral to secondary care whenever their respiratory symptoms remain unexplained. This was the case for at least 42 patients with a negative BCT result, since in the reports of their tests it was advised to consider referral to a pulmonologist because the diagnosis was unclear.

### Regression analysis

Using binary logistic regression, we analyzed whether there were any predictors of a positive BCT result among patients with probable and possible asthma. The cases are independent, the dependent variable is dichotomous, the data set is large (*n* = 645), and there is no multicollinearity between the predictors. There is a linear connection between independent variables and the log odds. The initial univariate analysis showed that the following variables were related to a positive BCT response with a *P* value < 0.10: age, positive family history, ever smoked, seasonal triggers, hyperreactivity for baking odors, allergy for pets, wheezing (ACQ 5), use of bronchodilators (ACQ 6), age of onset, reversibility, and the pre- and post-bronchodilator values for both FEV_1_ and FEV_1_/FVC (% predicted) (Supplementary Table 1). Therefore, these predictors were included in the log regression analysis (Table 3).

**Table 3:** Final regression model.

Variable	*B*	S.E.	Wald	df	Sig.	Exp(*B*)	95% CI for Exp(*B*)	
							Lower	Upper
Age of the patient	−0.03	0.01	13.70	1.00	0.00	0.97	0.96	0.99
Positive family history	0.31	0.17	3.28	1.00	0.07	1.37	0.98	1.91
Pet allergy	0.62	0.25	6.23	1.00	0.01	1.85	1.14	3.01
Frequency of bronchodilator use in the past week (ACQ6)	0.28	0.12	5.69	1.00	0.02	1.32	1.05	1.65
Reversibility in %	0.04	0.02	2.84	1.00	0.09	1.04	0.99	1.08
FEV_1_ pre in %	−0.02	0.01	4.66	1.00	0.03	0.99	0.97	1.00
FEV_1_/FVC post in %	−0.07	0.02	19.80	1.00	0.00	0.93	0.91	0.96
Constant	8.16	1.43	32.34	1.00	0.00	3481.36		

*ACQ* Asthma Control Questionnaire, *B* beta, *CI* confidence interval, *df* degrees of freedom, *FEV*_*1*_ forced expiratory volume in 1 s, *FVC* forced vital capacity, *S.E*. standard error, *sig*. significance.

The final logistic model showed that predictors having a self-reported pet allergy (odds ratio (OR): 1.85; 95% confidence interval (CI): 1.14–3.01) and using a bronchodilator (OR: 1.32; 95% CI: 1.05–1.65) were associated with an increased odds of a positive bronchodilator response. By contrast, predictors that reduced the odds of having a positive BCT result were being older (year) (OR: 0.97; 95% CI: 0.96–0.99) and having better lung function on both the pre-bronchodilator FEV_1_ (liter) (OR: 0.99; 95% CI: 0.97–1.00) and the post-bronchodilator FEV_1_/FVC (OR: 0.93; 95% CI: 0.91–0.96). The proportion of variation predicted by this model was 12.5% (Nagelkerke *R*-squared).

## Discussion

In a literature search, we found no research into the use of direct BCT as a diagnostic tool in primary care settings. Our analysis shows that BCT by the histamine provocation test can be performed safely and reliably in a primary care diagnostic center. It was notable that the odds of having a positive BCT were increased among younger patients, those with self-reported pet allergy, those who used bronchodilators, and those with poorer lung function. Looking at the of diagnoses made following BCT, both rejecting and confirming asthma, we propose that BCT in primary care has the potential to reduce the rates of both misdiagnosis and referral to secondary care.

Although research by Aaron et al. has recently brought the issue of asthma misdiagnosis to the fore, the underdiagnosis and overdiagnosis is an ongoing global problem^5,18–21^. Misdiagnosis may lead to inappropriate prescribing and an increased health care use. In patients with a high probability of asthma, we showed that adding a histamine provocation test to standard spirometry with bronchodilator response can increase diagnostic accuracy in primary care. The addition of a BCT in the primary care setting can help reject or confirm the diagnosis and may reduce misdiagnosis rates. Indeed, when using methacholine, direct BCT has a sensitivity of 98% for detecting asthma^22^. By contrast, Schneider et al. showed that the sensitivity of simple spirometry was only 29% for diagnosing airway obstruction in asthma^7^, and data showed that the sensitivity of primary care spirometry alone fell to just 16% when diagnosing asthma. Given that normal spirometry results do not rule out asthma, further investigation is needed in patients who otherwise present with characteristic respiratory symptoms.

In recent years, data have been published on the use of combined asthma–COPD services in primary care^23–27^. These services provide spirometry facilities, with all tests performed being assessed by a pulmonologist who then gives the GP a structured diagnostic and therapeutic assessment based on a short history and the spirometry results^27^. These services have proven helpful in diagnosing or excluding asthma, COPD, and combined asthma and COPD in primary care. Implementing BCT in these primary care diagnostic centers could further improve diagnostic accuracy and reduce overtreatment. Instead of starting a therapeutic trial of an ICS for 3 months, these centers could perform BCT to confirm or exclude the diagnosis. Based on real-life data from an asthma–COPD service in Groningen, the Netherlands, Metting et al. developed a diagnostic decision tree for assessing obstructive lung disease^28^. Applying this decision tree to our cohort of 645 patients would have resulted in 457 being diagnosed with asthma and receiving a therapeutic trial of ICS. However, only 272 of these patients had a positive BCT result, with the remaining 185 having a negative result. By integrating BCT, the risk of this overtreatment can be reduced while also ensuring that alternative diagnoses are not missed. Manoharan et al. also showed us that 30% of 123 patients with community managed asthma in Scotland were potentially misdiagnosed or overtreated as they were non-responsive to both methacholine and mannitol challenge testing^29^.

We encountered no adverse events in our cohort, demonstrating the safety of direct histamine provocation testing in this primary care diagnostic center in real-life conditions. This indicates that BCT can be safely implemented in other primary care settings if they are adequately equipped and staffed. The necessary safety precautions, as outlined by the American Thoracic Society/European Respiratory Society (ATS/ERS) guidelines, must be guaranteed. Research has already shown that the BCT can be used in primary care to monitor asthma treatment, with Turton et al. concluding that the mannitol challenge test was feasible and acceptable^30^. We therefore believe that there is growing evidence for current guidelines to be adapted. In our opinion, BCT does not necessarily have to take place in secondary care, it can now be recommended in well-equipped primary care diagnostic centers. Of course, this is contingent on ensuring that the indication for BCT is appropriate, that spirometry is normal, that necessary diagnostic symptoms and histories are available, and that the results are assessed by a professional who is experienced in interpreting pulmonary function tests (PFTs).

Current guidelines endorse referral for BCT to confirm or reject the diagnosis of asthma whenever doubt remains unclear after spirometry. In point of fact, positive and negative BCT results contribute equally to diagnostic accuracy in patients with respiratory symptoms^14,17^. Given these considerations, our data indicate that diagnostic referrals to secondary care could be dramatically reduced if more primary care diagnostic centers started to provide BCT. Only those patients with unexplained respiratory symptoms and a negative BCT result may still need referral to secondary care, though this may not necessarily include review by a pulmonologist. Other causes of respiratory symptoms such as chest tightness and cough must also be considered. Indeed, the GP who requested the PFTs must also consider other possible causes, such as gastro-esophageal reflux or post-nasal drip, or if they simply need to refer directly. Follow-up research should clarify whether patients from our real-life cohort remain labeled as non-asthmatic or are eventually labeled as having asthma when referred to secondary care.

This was only a retrospective analysis of a real-life patient cohort, and as such, we did not have all the data on patient characteristics for the total cohort of 998 patients. That said, we had all relevant data for the BCTs (i.e., results and side effects) and near-complete data for the cohort who underwent both spirometry and BCT. There was a notably good correlation when comparing the characteristics of this group to other respiratory patients in primary care^26,29,31^. All patients who underwent BCT at the primary care diagnostic center had normal spirometry results and were not initially referred to a pulmonologist by their GP. Therefore, the cohort typically comprised patients with relatively mild symptoms, which may have contributed to the lack of adverse events during BCT. Nevertheless, we should point out that there is a low incidence of adverse events with BCT in the available literature^8^.

At the time of assessment, some patients were already using inhaled medications due to the real-life design. In all cases, beta-agonists and anti-muscarinic agents were stopped 6–48 h before BCT, depending on their duration of action and the relevant ATS/ERS guidelines. Antihistaminic drugs were stopped at least 72 h before the test. Whenever a patient was using ICS, this was known to the assessor and was taken into consideration when interpreting the BCT results. Despite using an ICS, the majority of these patients (77% in total cohort and 56% in patients in the high probability asthma group) still had a positive BCT result. BHR to histamine does not normalize when using ICS, at most the level of severity may decrease by two doublings of the geometric mean PD_20_^32,33^. As we use a cut-off of >2.39 mg histamine to exclude BHR (corresponding with a provocative concentration causing a 20% fall in FEV_1_ (PC_20_) of 16 mg/mL), patients on ICS with a negative BCT could almost certainly be labeled as not having asthma. Nonetheless, in specific cases, when there was a notable FEV_1_ reduction it was advised to repeat BCT after stopping ICS for at least 4 weeks.

A small part of the final cohort (7%) showed airflow obstruction post BD (FEV_1_/FVC ≤70%); in the high probability asthma group, this was 4%. Despite this fixed obstruction, not all of these patients showed BHR when tested. In these patients, the differential diagnosis should be reconsidered, as there is evidence of airway pathology. In these cases, it was recommended to repeat BCT after at least 4 weeks (if applicable, after stopping inhalation medication) or to refer to a pulmonologist.

Diagnosing asthma is challenging. Despite its utility, we acknowledge that a BCT in a general population is not a gold standard test for diagnosing or excluding asthma. The specificity and sensitivity of a direct BCT for diagnosing asthma is strongly dependent on the population being tested^8,17^. Perpiñá et al. estimated that the optimal diagnostic value of methacholine challenge testing occurs when the pretest probability of asthma is 30–70%^34^. In our final cohort, we only selected patients with a high pretest probability of asthma. Consequently, in this selected group with respiratory symptoms and suspected of asthma we used the histamine provocation test as a tool to confirm or reject asthma. Furthermore, direct challenge tests generally have a good sensitivity to exclude current asthma in patients with respiratory symptoms. Whether a patient had symptoms at the time of the test was known to the assessor and was considered when assessing the BCT results.

To date, the best combination of clinical features and diagnostic tests has not been found. An alternative might be to use the exhaled nitric oxide (FeNO) test, which can also contribute to an accurate diagnosis of asthma^35^. Although there are no reports of the FeNO test being used in primary care settings, if the BCT could be routinely implemented with success, there may also be a valid argument for implementing the FeNO test. Further research is warranted on this point.

The real-life retrospective study design meant that we lacked a control group against which to compare our results. If a randomized controlled trial is performed to compare BCT in primary and secondary care, we predict this will reach similar conclusions with regards the safety, feasibility, and diagnostic accuracy. Nevertheless, the strength afforded by prospective research would reinforce the argument that BCT has a role in primary care settings. Prospective research should ideally follow the group of patients with respiratory symptoms, normal spirometry, and a negative BCT who are referred to secondary care to record the additional diagnostic steps and the final diagnosis. To assess whether diagnostic referrals can be reduced, a randomized trial should prospectively compare a cohort who undergo BCT in primary care and another who are referred directly to secondary care.

Our data in a real-life primary care setting indicate that histamine provocation testing is feasible and safe. In our cohort, we believe it improved the accuracy of asthma diagnosis. Consequently, using BCT in primary care, in addition to spirometry alone, has the potential to reduce the rates of asthma misdiagnosis and the need for diagnostic referrals to secondary care. We therefore recommend that the use of a BCT be considered in well-equipped primary care laboratories as it provides a valuable addition to spirometry with bronchodilator response in the diagnostic work-up of patients with respiratory symptoms.

## Methods

### Study population

We conducted a real-life observational study at the Star-shl primary care diagnostic center, Rotterdam, the Netherlands. GPs were free to refer patients for diagnostic tests, including PFT. The center is licensed to carry out spirometry with bronchodilator response and direct BCT as histamine provocation testing. All PFTs are performed by qualified and certified pulmonary function technologists, and the results are assessed by a consulting pulmonologist or a GP with special interest in respiratory medicine. We retrospectively analyzed data for all patients with respiratory symptoms referred for diagnostic assessment by their GP between 2012 and 2017 if they were aged ≥16 years and underwent a histamine provocation test. The patients were required to have respiratory symptoms consistent with asthma in their differential diagnosis but without significant airflow reversibility demonstrated by prior testing. Airflow reversibility was defined as a change of ≥200 mL and a ≥12% FEV_1_ post BD.

In this study, we used anonymous assessment data. According to Dutch regulations, a separate ethics committee approval and informed consent from human participants is not required, because routinely collected health care data are used after anonymization.

### Respiratory assessment at the primary care diagnostic center

**Fig. 2:**
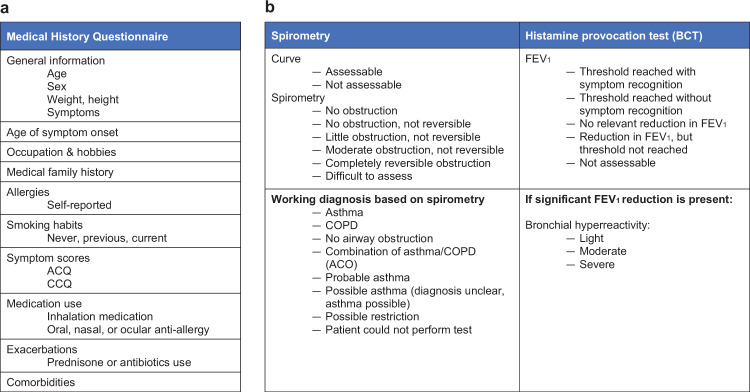
Questionnaire items and structure of the PFT assessment. **a** Items on questionnaire before spirometry. **b** Format for structured PFT assessment. ACQ Asthma Control Questionnaire, COPD Control Questionnaire, ACO asthma/COPD overlap, FEV_1_ forced expiratory volume in 1 s.

At their first visit, spirometry was performed with bronchodilator responsiveness according to ATS/ERS standard procedures^36^. All patients who underwent spirometry were asked to complete a structured questionnaire covering relevant clinical information (Fig. 2a), including the ACQ, and the consulting pulmonologist or GP is provided with these details and the spirometry results. Diagnostic assessment was then reported on a standardized protocol (Fig. 2b), though comments could be added if necessary, and the requesting GP was given a report of the assessment. Wherever possible, a working diagnosis and therapeutic or diagnostic recommendations (e.g., to perform BCT) were given without seeing the patient. The following working diagnoses were given based on the spirometry results: asthma, COPD, no airway obstruction, combined asthma and COPD, probable asthma, possible asthma, possible restriction, and patient could not perform test (Fig. 2b). “Probable asthma” referred to patients with a typical history of asthma, but whose spirometry results were not diagnostic. “Possible asthma” referred to patients without a typical history of asthma, but whose spirometry results and symptoms suggested that asthma remained possible (Fig. 1).

### Bronchial challenge testing

BCT was performed by histamine provocation test. The primary endpoint was a PD_20_, according to the most recent ERS guideline^8^. The histamine provocation test was only performed in patients who had already undergone spirometry testing either at the diagnostic center or elsewhere. BCT results were also assessed by an consulting GP or pulmonologist. The assessment takes place chronologically; the assessor first assessed the spirometry results; at a later time the BCT was assessed based on the test results and short medical history and spirometry results from the first visit.

The histamine dose itself was delivered using a 2-min tidal breathing protocol, with a computer-controlled nebulizer (APS Pro for the JAEGER® MasterScreen PFT Series) guaranteeing a reproducible inhaled dose and a high-efficiency filter to eliminate the risk of cross-contamination. The patients’ breathing pattern was displayed on a flow/time diagram to control challenge substance inhalation within 2 min. The histamine concentration was 32 mg/mL and delivered at a starting dose of 0.05 mg, with doses doubled in a stepwise manner based on flow/volume curves to a maximum dose of 3.1 mg. Post-diluent FEV_1_ was assessed at 30 s and at 90 s after completing nebulization. The level of bronchial hyperreactivity is then classified by the provocative histamine dose: severe, <0.15 mg; moderate, 0.15–0.60 mg; mild, 0.60–2.39 mg; and normal, >2.39 mg (Fig. 2a). If the FEV_1_ declined ≥20%, we administered 400 μg salbutamol via a metered-dose inhaler with a valved holding chamber. If the FEV_1_ failed to improve to ≥90% of the baseline FEV_1_, we first gave an extra 200 μg salbutamol. If this was inadequate, we also gave 40 μg ipratropium. The patient’s GP was informed, before discharge, in all cases where the FEV_1_ did not return to ≥90% of the baseline level. If patients experienced symptoms and the FEV_1_ did not decline, they were still given 400 μg of salbutamol.

Possible issues with test safety were mitigated by following the relevant ATS/ERS guidelines. When performing BCT, a medical doctor was always present in the building, and oxygen and medication were available in the testing room to treat any cases of severe bronchoconstriction.

### Feasibility, safety, and diagnostic value

A feasibility analysis was conducted in which we retrospectively looked at the number of referrals, the correct indication of these referrals, the number of BCTs carried out per year, and the quality of BCTs during the studied 5-year period.

To analyze patients with a high probability of asthma, we only included patients who performed a spirometry test at our diagnostic center before undergoing BCT: the final cohort. This improved the chance of obtaining consistent high-quality baseline spirometry results and working diagnoses based on expert assessment. Furthermore, BCT was usually advised by the expert assessor, so the indication for the test was assumed to be correct. Patients labeled as probable or possible asthma were included because these had a high pretest probability of asthma. We calculated how often BCT was positive, thereby confirming the diagnosis of asthma.

Finally, we assessed the safety of BCT in the primary care diagnostic center by looking at the adverse events during the test and that were reported back to us after performing the test.

### Statistical analysis

IBM SPSS Version 22 (IBM Corp., Armonk, NY, USA) was used for statistical analysis. The baseline population characteristics were described by descriptive statistics. We compared the baseline characteristics and outcomes by *t* test or Mann–Whitney *U* test for continuous outcomes and *χ*^2^ test for nominal outcomes. We used individual univariate regression analysis to determine the relevant predictors for inclusion in the logistic regression and included only those variables with a *P* value < 0.1. Binary backward logistic regression was used to determine the characteristics predicting a positive BCT test. Likelihood ratio tests were used for the backward analyses. The raw values, ORs, and 95% CIs are presented, as appropriate.

### Reporting summary

Further information on research design is available in the Nature Research Reporting Summary linked to this article.

## Supplementary information

Supplementary Table 1

Reporting Summary

## Data Availability

Due to the Dutch legislation and the General Data Protection Regulation, it is not possible to make the dataset available because they were collected for clinical purposes. Data were not collected for research purposes, this is real-life data. Patients were able to opt out before their assessment. It is, however, possible to send a request for access to the acronymized data via the research steering committee of the laboratory. However, the output of the analyses and the protocols are available for other researchers and readers.
