# Supplementary material for: The use of a direct bronchial challenge test in primary care to diagnose asthma
Source: NPJ Prim Care Respir Med. 2020 Oct 16;30:45. doi: 10.1038/s41533-020-00202-y (PMC7567813; doi:10.1038/s41533-020-00202-y)
Supplement: Supplementary file 1 — Supplementary Table 1 [file 41533_2020_202_MOESM1_ESM.pdf]

Supplementary Table 1. Parameter estimates

Dependent Variable:

Parameter	B	Std. Error	t	Sig.	95% Confidence Interval Lower	Upper	Partial Eta Squared
Intercept	0.592	0.024	24.390	0.000	0.544	0.639	0.481
[Gender=0]	-0.025	0.041	-0.609	0.543	-0.104	0.055	0.001
[Gender=1]	0[a]						
Intercept	0.679	0.06	11.375	0	0.562	0.796	0.168
Age.1	-0.002	0.001	-1.702	0.089	-0.005	0	0.004
Intercept	0.626	0.030	21.188	0.000	0.568	0.684	0.416
[fam.categ=.00]	-0.079	0.039	-2.013	0.044	-0.157	-0.002	0.006
[fam.categ=1.00]	0[a]						
Intercept	0.416	0.100	4.162	0.000	0.220	0.612	0.026
BMI.1	0.006	0.004	1.701	0.089	-0.001	0.013	0.004
Intercept	0.622	0.029	21.368	0.000	0.565	0.680	0.415
[Smoked.1=.00]	-0.071	0.039	-1.815	0.070	-0.148	0.006	0.005
[Smoked.1=1.00]	0[a]						
Intercept	0.589	0.036	16.514	0.000	0.519	0.659	0.298
[ICS.1=.00]	-0.008	0.043	-0.187	0.851	-0.091	0.076	0.000
[ICS.1=1.00]	0[a]						
Intercept	0.511	0.053	9.730	0.000	0.408	0.615	0.128
[All_NONE=.00]	0.083	0.057	1.466	0.143	-0.028	0.194	0.003
[All_NONE=1.00]	0[a]						
Intercept	0.640	0.039	16.474	0.000	0.563	0.716	0.297
[All_ever.SEIZOENEN=.00]	-0.076	0.045	-1.689	0.092	-0.164	0.012	0.004
[All_ever.SEIZOENEN=1.00]	0[a]						
Intercept	0.609	0.051	11.825	0.000	0.508	0.710	0.179
[All_ever.BOMEN=.00]	-0.030	0.056	-0.540	0.589	-0.139	0.079	0.000
[All_ever.BOMEN=1.00]	0[a]						
Intercept	0.622	0.081	7.658	0.000	0.462	0.781	0.084
[All_ever.VOEDSEL=.00]	-0.041	0.084	-0.491	0.624	-0.205	0.123	0.000
[All_ever.VOEDSEL=1.00]	0[a]						
Intercept	0.441	0.084	5.221	0.000	0.275	0.607	0.041
[All_ever.BAKLUCHT=.00]	0.150	0.087	1.724	0.085	-0.021	0.320	0.005
[All_ever.BAKLUCHT=1.00]	0[a]						
Intercept	0.601	0.039	15.308	0.000	0.524	0.678	0.267
[All_ever.KOUDELUCHT=.00]	-0.024	0.045	-0.537	0.592	-0.113	0.065	0.000
[All_ever.KOUDELUCHT=1.00]	0[a]						
Intercept	0.587	0.047	12.413	0.000	0.494	0.680	0.193
[All_ever.GRASSEN=.00]	-0.005	0.052	-0.098	0.922	-0.107	0.097	0.000
[All_ever.GRASSEN=1.00]	0[a]						

Intercept	0.702	0.048	14.577	0.000	0.607	0.796	0.248
[All_ever.HUISDIEREN=.00]	-0.142	0.053	-2.698	0.007	-0.245	-0.039	0.011
[All_ever.HUISDIEREN=1.00]	0[a]						
Intercept	0.610	0.032	18.802	0.000	0.547	0.674	0.355
[All_ever.STOF=.00]	-0.043	0.041	-1.055	0.292	-0.122	0.037	0.002
[All_ever.STOF=1.00]	0[a]						
Intercept	0.549	0.045	12.290	0.000	0.461	0.637	0.190
[All_ever.ANDERS=.00]	0.042	0.050	0.839	0.402	-0.056	0.139	0.001
[All_ever.ANDERS=1.00]	0[a]						
Intercept	0.556	0.028	19.944	0.000	0.501	0.611	0.382
[All_ever.INSPANNING=.00]	0.053	0.039	1.352	0.177	-0.024	0.129	0.003
[All_ever.INSPANNING=1.00]	0[a]						
Intercept	0.564	0.067	8.465	0.000	0.433	0.694	0.100
[Dich_Nasal.antiallergy.1=.00]	0.021	0.070	0.303	0.762	-0.116	0.158	0.000
[Dich_Nasal.antiallergy.1=1.00]	0[a]						
Intercept	0.568	0.055	10.350	0.000	0.460	0.676	0.143
[Dich_Oralallergy.1=.00]	0.017	0.059	0.293	0.769	-0.098	0.132	0.000
[Dich_Oralallergy.1=1.00]	0[a]						
Intercept	0.584	0.026	22.683	0.000	0.533	0.634	0.445
ACQ1.1	0.000	0.014	-0.036	0.972	-0.028	0.027	0.000
Intercept	0.613	0.028	21.620	0.000	0.557	0.669	0.421
ACQ2.1	-0.020	0.014	-1.458	0.145	-0.048	0.007	0.003
Intercept	0.606	0.033	18.107	0.000	0.541	0.672	0.338
ACQ3.1	-0.013	0.015	-0.859	0.391	-0.044	0.017	0.001
Intercept	0.602	0.036	16.674	0.000	0.531	0.673	0.302
ACQ4.1	-0.009	0.015	-0.631	0.528	-0.039	0.020	0.001
Intercept	0.548	0.027	20.373	0.000	0.495	0.601	0.392
ACQ5.1 wheezing	0.026	0.014	1.875	0.061	-0.001	0.054	0.005
Intercept	0.557	0.022	25.776	0.000	0.515	0.600	0.508
ACQ6.1 use of bronchodilators	0.065	0.024	2.669	0.008	0.017	0.113	0.011
Intercept	0.579	0.035	16.339	0.000	0.509	0.649	0.293
ACQ_total.1	0.003	0.022	0.130	0.896	-0.040	0.045	0.000
Intercept	0.650	0.041	16.010	0.000	0.570	0.729	0.289
Ageofonset.1	-0.002	0.001	-1.942	0.053	-0.004	0.000E+00	0.006
Intercept	0.532	0.026	20.369	0.000	0.480	0.583	0.397
Reversibility in %	0.014	0.005	2.861	0.004	0.004	0.024	0.013
Intercept	1.279	0.203	6.288	0.000	0.880	1.679	0.058
fev1_fvc Post	-0.009	0.003	-3.439	0.001	-0.014	-0.004	0.018
Intercept	1.312	0.197	6.679	0.000	0.927	1.698	0.066
fev1_fvc Pre	-0.009	0.002	-3.739	0.000	-0.014	-0.004	0.022
Intercept	1.138	0.146	7.782	0.000	0.851	1.426	0.086

fev1Post % of predicted	-0.006	0.001	-3.824	0.000	-0.008	-0.003	0.022
Intercept	1.244	0.143	8.673	0.000	0.963	1.526	0.107
fev1Pre % of predicted	-0.007	0.001	-4.657	0.000	-0.010	-0.004	0.033
Intercept	0.665	0.148	4.485	0.000	0.374	0.956	0.030
fvcPost % of predicted	-0.001	0.001	-0.552	0.581	-0.004	0.002	0.000
Intercept	0.697	0.148	4.727	0.000	0.408	0.987	0.034
fvcPre % of predicted	-0.001	0.001	-0.786	0.432	-0.004	0.002	0.001

Abbreviations: *ACQ* asthma control questionnaire, *BMI* body mass index, *FEV1* forced expiratory volume in 1 s, *FVC* forced vital capacity, *ICS* inhalation corticosteroid

LEGEND

All.none - no allergy

All.ever - Allergy ever for (*Seizoenen* seasons; *Bomen* trees; *Voedsel* nutrition; *Baklucht* baking air; *Koude lucht* cold air; *Grassen* grass; *Huisdieren* pets; *Stof* dust; *Anders* other; *Inspanning* exercise;

Dich_Oralallergy – takes oral anti-allergy medication

Dich_Nasal.antiallergy – takes nasal anti-allergy medication

fam.categ - positive family history for asthma

ICS – inhaled corticosteroid use
